# Modeling the effect of copper availability on bacterial denitrification

**DOI:** 10.1002/mbo3.111

**Published:** 2013-07-30

**Authors:** Hugh C Woolfenden, Andrew J Gates, Chris Bocking, Mark G Blyth, David J Richardson, Vincent Moulton

**Affiliations:** 1 School of Computing Sciences, University of East AngliaNorwich Research Park, Norwich, NR4 7TJ, U.K; 2 School of Biological Sciences, University of East AngliaNorwich Research Park, Norwich, NR4 7TJ, U.K; 3 School of Mathematics, University of East AngliaNorwich Research Park, Norwich, NR4 7TJ, U.K

**Keywords:** Bioreactor, Michaelis–Menten kinetics, nitrous oxide, *Paracoccus denitrificans*, reductases, respiratory model

## Abstract

When denitrifying bacteria such as *Paracoccus denitrificans* respire anaerobically they convert nitrate to dinitrogen gas via a pathway which includes the potent greenhouse gas, nitrous oxide (N_2_O). The copper-dependent enzyme Nitrous Oxide reductase (Nos) catalyzes the reduction of N_2_O to dinitrogen. In low-copper conditions, recent experiments in chemostats have demonstrated that Nos efficiency decreases resulting in significant N_2_O emissions. For the first time, a chemostat-based mathematical model is developed that describes the anaerobic denitrification pathway based on Michaelis–Menten kinetics and published kinetic parameters. The model predicts steady-state enzyme levels from experimental data. For low copper concentrations, the predicted Nos level is significantly reduced, whereas the levels for the non copper-dependent reductases in the pathway remain relatively unaffected. The model provides time courses for the pathway metabolites that accurately reflect previously published experimental data. In the absence of experimental data purely predictive analyses can also be readily performed by calculating the relative Nos level directly from the copper concentration. Here, the model quantitatively estimates the increasing level of emitted N_2_O as the copper level decreases.

## Introduction

Nitrous oxide (N_2_O) is the third largest contributor to global warming behind carbon dioxide (CO_2_) and methane. The emission of N_2_O significantly affects the rate of global warming because it is a greenhouse gas and due to its destructive effect on ozone. Indeed, a molecule of N_2_O is around 300 times more potent a greenhouse gas than CO_2_ (IPCC 2007, p. 212). Agriculture is responsible for the majority of N_2_O emissions largely due to the use of nitrate-based fertilizer. Under low oxygen conditions denitrifying soil bacteria, with the ability to respire anaerobically, can reduce nitrate to dinitrogen via nitrite, nitric oxide and N_2_O in a series of sequential reactions. Depending on the soil type, bacterial populations present and other factors the end product of denitrification may be emission of either N_2_O or N_2_. It is therefore important to develop predictive models of denitrification to provide accurate emission estimates for the constituents of the pathway, in particular N_2_O.

Previous work on modeling denitrification considers the full range of spatial and temporal scales. The review by Heinen (2006) covers over 50 models that address a wide variety of systems including soil, sediment and whole terrestrial ecosystems. When generating the time evolution in these models the time steps can be of the order of 1 month and the denitrification module parameterized by a single rate. While these models integrate a large number of parameters, the systems they seek to describe are nonetheless governed by a plethora of bacterial species. Therefore, the models are approximations to the reaction kinetics because the actual mechanism may only be partially understood, or the bioavailability of essential minerals, for example iron and copper, omitted entirely.

The processes by which microbes emit and consume N_2_O have been reviewed by Richardson et al. (2009). Specific enzymes in the denitrification pathway have also been the subject of detailed biochemical studies (e.g., Field et al. 2008) together with the effect exerted by genetic regulation (e.g., Bergaust et al. 2012). However, this wealth of information regarding the enzymes, and specifically their kinetic behavior, has yet to be integrated into a robust mathematical model of the chemical reactions. For example, models based on Michaelis–Menten kinetics (e.g., Cornish-Bowden 2012) have been used to supplement experimental work (e.g., Betlach and Tiedje 1981; Xu and Enfors 1996), but in most cases model parameters were chosen arbitrarily and the models used to qualitatively explain their experimental observations. By contrast, other models (e.g., Thomsen et al. 1994) calculate the kinetic parameters so that the model fits their experimental results. However, the models were not used to make more general quantitative predictions regarding the intermediates in the denitrification pathway.

Here we develop a model of denitrification for the intensively studied bacterium, *Paracoccus denitrificans*, for which many of the kinetic parameters in the denitrification pathway are known. Moreover, it has been recently shown that under certain conditions *P. denitrificans* will emit significant amounts of N_2_O when copper is scarce (Felgate et al. 2012), permitting us to quantitatively model this effect for the first time. In *P. denitrificans*, the following reactions constitute the anaerobic denitrification pathway:


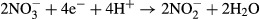
(1)


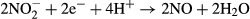
(2)


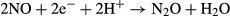
(3)


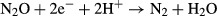
(4)

where reactions (1)–(4) are catalyzed by Nitrate reductase (Nar), Nitrite reductase (Nir), Nitric Oxide reductase (Nor), and Nitrous Oxide reductase (Nos), respectively. These reactions consume a total of 10 electrons and 12 protons when two 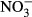 ions are converted to a single molecule of N_2_ gas. Therefore, both electrons and protons must be available if the reductases are to effectively catalyze the reactions. Furthermore, the ability of *P. denitrificans* to successfully reduce 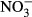 to N_2_ hinges on the existence of copper-dependent Nos which is a homodimeric holoenzyme that binds six copper atoms per monomer (Brown et al. 2000). The copper requirement for Nos enzyme activation can be viewed as:


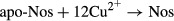
(5)

where apo–Nos is the inactive pro-protein prior to copper insertion. It should be noted that there are several forms of the Nos enzyme each with different catalytic activities (Zumft 1997; Table 9; Rasmussen et al. 2002). Here, all references to Nos refer to the most catalytically active form unless stated otherwise.

Following Betlach and Tiedje (1981) and Xu and Enfors (1996) we treat reactions (1)—(4) as a series of enzyme-substrate reactions and apply Michaelis–Menten kinetics. We write the reactions as:


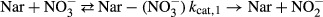
(1a)


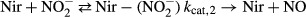
(2a)


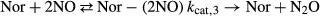
(3a)


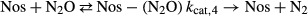
(4a)

where, for brevity, only the metabolites and enzymes are retained. The Michaelis constant and limiting rate for reaction *i* are K_M,*i*_ and *V*_max,*i*_, with their referenced experimentally derived values shown in Table S1. A mass balance for each metabolite leads to the set of differential equations shown in Table 1. By seeking the steady-state solution, expressions that estimate the reductase concentrations are obtained. Using these values, time courses for 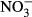 and other relevant metabolites can then be calculated. The reductase levels obtained from the low- and high-copper experiments thus provide enzyme levels for further predictive analyses. The equations are shown in Table 1 and the formulation detailed in the next section.

**Table 1:** Equations and parameters for the model

Rate equations		
d*n*_*i*_ /d*t* = *M*_*i*-1_−*s*_*i*_ *M*_*i*_ + D (*n*_*i*,In_−*n*_*i*_ )	*i* = 1…5	(5)
M_0_ = M_5_ = 0, M_*i*_(*n*_*i*_) = *V*_max,*i*_*n*_*i*_ /(*K*_M,*i*_ + *n*_*i*_ )	*i* = 1…4	
Implied experimental enzyme concentrations		
e_*i*_ = (1 + K_M*i*_ /*n*_*i*_)(M_*i*−1_ + D (*n*_*i*,In_−*n*_*i*_) − d*n*_i_ /d*t*)/(s_*i*_ k_cat*i*_)	*i* = 1…4	(6)
Implied steady-state enzyme concentrations		
e_i,ss_ = (1 + K_M*i*_ /*n*_*i*_)(M_*i*−1_ + D (*n*_*i*,In_−*n*_*i*_)) / (s_*i*_ k_cat *i*_)	*i* = 1…4	(7)
Predicted Nos concentration		
e_4,ss_([Cu]) = e_4,Init_([Cu]) = *α*[Cu]/(β + [Cu])		(8)
e_4_(*t*) = e_4,Init_ exp(−Dt) + e_4,ss_ (1–exp(−Dt))		(9)
Nomenclature		
*n*_1_ = [ 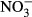 ], *n*_2_ = [ 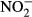 ], *n*_3_ = [NO], *n*_4_ = [N_2_O] and *n*_5_ = [N_2_]		
e_1_ = [Nar], e_2_ = [Nir], e_3_ = [Nor] and e_4_ = [Nos]		

Parameter	Description	Value
s_*i*_	Substrate stoichiometric constant	2 for *i* = 3, else 1
K_M,*i*_	Michaelis constant for reaction *i*	Table S1
*V*_*max,i*_ (= k_cat,i_ e_i_)	Limiting rate for reaction *i*	
k_cat,*i*_	Turnover number for reaction *i*	Table S1
D	Dilution rate	0.05 h^−1^
*n*_*i*,In_	Inflow concentration for *n*_*i*_	20 mmol/L for *i* = 1 0 mmol/L for *i* > 1
α, β	Calibration parameters	Table S3

For a given experiment the model therefore provides a method of predicting the enzyme concentrations and the time courses for the metabolites. The model also facilitates the quantitative prediction of N_2_O emissions (and the other metabolites) for a prescribed copper concentration.

The model is available for download from http://www.uea.ac.uk/computing/software/modelling-denitrification.

## Experimental Procedures

### Model

In a chemostat of volume, *V*, the overall rate of change in the concentration in the vessel, *n*, is the sum of the rate of transformation and the rate of transport. The latter is governed by the flow rate, *F*, which sets the rate at which substrate enters and leaves the vessel. Mathematically the overall rate of change in *n* can be expressed as:


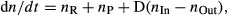


where *n*_R_ ≤ 0 is the rate of reduction, *n*_P_ ≥ 0 is the rate of production, *D* = *F*/*V* is the dilution rate, *n*_In_ is the inflow concentration, and *n*_Out_ is the outflow concentration. Here we assume that *n*_In_ is constant and the vessel contents are well-mixed so that *n*_Out_ = *n*. The solution to the above equation provides the time evolution of n and may be found provided the reaction rates, *n*_R_ and *n*_P_, are known together with *n*_In_ and the initial concentration. We use Michaelis–Menten kinetics to obtain formulae for *n*_R_ and *n*_P_ and apply to reactions (1a)–(4a) to yield the overall rate equations (5) given in Table 1. The model assumes 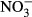 is the only nitrogenous compound fed into the chemostat, as was the case for the experiments of Felgate et al. (2012). The model could be easily updated if cells were grown in the presence of either 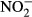 or N_2_O as sole respiratory substrate. An appropriate ordinary differential equations (ODEs) solver can be used to obtain the solution to equations (5) given the initial conditions and the kinetic constants. In the derivation we consider each reaction to be first order with respect to the substrate (e.g., Betlach and Tiedje 1981). Although equations (5) are similar to those given in Betlach and Tiedje (1981) and Xu and Enfors (1996), the equations given here are more general because the dilution rate is included. However neither study presented the overall rate for each step in the pathway. The stoichiometry of the nitric oxide reduction is reflected in the equation for d*n*_3_/d*t* by the coefficient of 2 on the right-hand side. Furthermore, this coefficient ensures the overall N-mass balance.

When coupled with experimentally obtained concentrations for the nitrogenous compounds, equations (5) can be rearranged to obtain equations (6) which predict the enzyme levels. The equations can be used to calculate enzyme concentrations throughout the time course. However, values for the time derivatives would need to be approximated from the experimental data. It is important to point out that the formulae for the implied concentrations are derived on the assumption of homogeneity in the chemostat. Since the enzymes only occur within the bacteria, the volume fraction of the chemostat occupied by *P. denitrificans* cells is implicitly included in each concentration value. This does not affect the mathematical model provided the implied concentrations are used consistently. However, when discussing relative levels of each respiratory enzyme we divide the concentrations by the concentration for Nar thereby removing the volume fraction factor. The implied steady-state enzyme concentrations, labeled *e*_i,ss_ (for enzyme *i*) are given in equation (7), and are obtained from equation (6) by setting the time derivative to zero. These will, in all likelihood, differ to those at an earlier time. Indeed, the time evolution of the expression of the genes for the reductases of *P. denitrificans* were investigated by Baumann et al. (1996) who showed that they peaked during the very early stages of anoxia before declining to a steady state. Here we will assume that the Nar, Nir and Nor concentrations are constant and do not change from their steady-state values. However, we do allow the Nos concentration to vary with time and this is discussed further in the next section.

### Calibration of the predicted Nos level

There are two experimental observations that must be incorporated into the model. The first is the vital impact of copper on the formation of the Nos enzyme. The second is the N_2_O emission lag observed in the low-copper Felgate et al. (2012) experiment.

There are several methods of modeling the Nos activation given in reaction (20) (e.g., Cornish-Bowden 2012; §6.7). Two such methods are essential activation and mixed activation. However, conceptually, there are problems with both of these schemes in relation to the copper insertion into the Nos pro-protein. Essential activation leads to an adjustment of the Michaelis constant and not the Nos level. Clearly, when copper falls below some critical level a lower amount of Nos is expressed because more N_2_O is emitted (Felgate et al. 2012). Mixed activation does affect the enzyme level but assumes that copper can be removed from Nos as well as inserted into apo–Nos. There is currently no evidence to support this. We therefore use a mathematical function to estimate the Nos level directly from the copper concentration and calibrate it using the implied Nos concentrations.

In order to select the mathematical form of the dependence between the copper level and the Nos level, we look at N_2_O production rates. The rates for high-copper ([Cu] = 13 μmol/L) and low-copper ([Cu] = 0.5 μmol/L) experiments involving *P. denitrificans* are quoted in Table 1 of Felgate et al. (2012), with the low-copper experiment having a dramatically larger rate. The low rate (<0.1 μmol N g^−1^ h^−1^) for the high-copper experiment shows that the Nos level is sufficient to reduce N_2_O to N_2_, and that the Nos concentration is possibly at (or is close to) a saturated level with respect to the intracellular copper concentration. The sharp increase in production rate (1200 μmol N g^−1^ h^−1^) in the low-copper experiment implies that the level of active Nos has dropped considerably. However, in both experiments the N_2_ production rates are significant showing that the Nos levels are nonzero. When copper is absent, N_2_O production is close to that observed for a *P. denitrificans* strain deficient in *nosZ* (D. J. Richardson, H. Felgate and G. Giannopoulos, unpublished result). Furthermore, N_2_ production is zero, which is consistent with a total absence of Nos. These three copper levels indicate that the Nos level changes rapidly as the copper level increases from zero, followed by a transition to eventual saturation. The observed copper dependence on N_2_O reduction, and thus the predicted Nos level, *e*_4,ss_, can therefore be described by a rectangular hyperbola (eq. 8 in Table 1) and calibrated using the Nos levels in Table 2. To perform error analysis, we require upper and lower bounds for *e*_4,ss_. Since these bounds will be used to measure changes due to the kinetic parameters, we calibrate them from the Nos concentrations corresponding to experimental error only. These Nos concentrations lie in the ranges 3.09–3.57 nmol/L and 0.19–0.25 nmol/L for the high-copper and low-copper experiments, respectively. The calibration constants are given in Table S3. As mentioned previously the bioavailability of copper in the low-copper experiment of Felgate et al. (2012) was reduced by the addition of ascorbate and BCA. Since the effective copper concentration is unknown, we use [Cu] = 0.5 μmol/L for calibration purposes and use it to represent the copper-limited case.

**Table 2:** Implied enzyme concentrations for the experiments of Felgate et al. (2012), together with the relative expression levels (RELs), their maximum variability and the ranges common to both copper levels

Experiment/Enzyme	Conc. (nmol/L)	Min. REL	REL	Max. REL
High copper (13 μmol/L)				
Nar	0.74	0.8	1.0	1.3
Nir	5.97	5.1	8.0	13.3
Nor	6.35	4.8	8.5	16.0
Nos	3.32	2.8	4.5	7.1
Low copper (0.5 μmol/L)				
Nar	1.05	0.8	1.0	1.4
Nir	2.87	2.1	2.7	3.8
Nor	8.90	4.6	8.5	16.6
Nos	0.23	0.1	0.2	0.3
Overlapping ranges				
Nar	0.80–0.97 nmol/L			
Nir	3.79–3.93 nmol/L			
Nor	4.80–11.88 nmol/L			
Nos	No overlap			

Equation (7) is used to calculate the implied concentration.

We chose to model the N_2_O emission lag by ascribing a time dependence to the Nos concentration. The initial level of Nos (labeled *e*_4,init_) is calculated from the experimental data. This approach mimics a process whereby a sufficient quantity of catalytically competent Nos forms at the onset of anoxia before being washed out of the chemostat to be replaced by less catalytically able Nos. The same hyperbola is used for *e*_4,Init_ although it is calibrated by treating the first N_2_O concentration in the anoxic period as the steady-state value. The calculation produces an implied Nos concentration of 0.77 nmol/L and the calibration constants are given in Table S3. Finally, the transition from the initial to the steady-state concentration is achieved by equation (9) in Table 1.

## Results

The model is calibrated from the nitrate-sufficient carbon-limited *P. denitrificans* results of (Felgate et al. 2012) because they result in the most significant emissions of N_2_O. The experiments were performed in a chemostat with a dilution rate of 0.05 h^−1^, an influent nitrate concentration of 20 mmol/L and copper concentrations of 0.5 μmol/L and 13 μmol/L. To ensure that copper availability was limited in the low-copper experiment, ascorbate and BCA were added to the medium to chelate extracellular copper. As we are interested in anaerobic denitrification we only consider time points after 24.5 h, which correspond to the anoxic period. Nitric oxide was not observed above the detection limit of 10 μmol/L, so we set a value of [NO] = 5 μmol/L (half the detectable amount) in all calculations. We also consider the system to be in steady state as per the time ranges given in Felgate et al. (2012) and all points falling in those ranges are pooled to calculate final concentrations and standard errors (values given in Table S2). The pooled values are assigned a time value of 130 h (for data handling purposes), which lies after the experimental points.

The results are divided into two main sections. First, we calculate the predicted enzyme levels for the two experiments and compute ranges suitable for predictive analyses. Here, experimental time courses are compared with those predicted by the model. Then we provide several predictive analyses for a range of copper concentrations to highlight the model's sensitivity to the copper level.

### Application to experiments

First, we use the model to obtain predictions for the denitrification enzyme concentrations before using those levels to calibrate the calculation of the time courses. We calculate the implied steady-state enzyme concentrations for the high- and low-copper experiments using equation (7) in Table 1. The values are shown in Table 2 together with the expression level relative to the Nar concentration. The maximum variability in the implied concentrations was found by recalculating the values after adjusting both the kinetic constants and the experimental end-points to the limit of their error bounds. Comparison of the predicted enzyme levels for Nar, Nir, and Nor reveals that in each case there is an overlapping range of concentrations (given in Table 2). This is consistent with the levels of Nar, Nir, and Nor being relatively insensitive to the copper concentration. However, we are unable to identify a range for the Nos concentration because the implied levels differ by an order of magnitude, that is, the Nos concentration is ∼ 10–30 times greater in the high-copper experiment than in the low-copper experiment. These disparate values emphasize how heavily Nos expression depends on copper and highlight the vital importance of including an explicit copper dependence when modeling the denitrification pathway in *P. denitrificans*.

**Figure 1:**
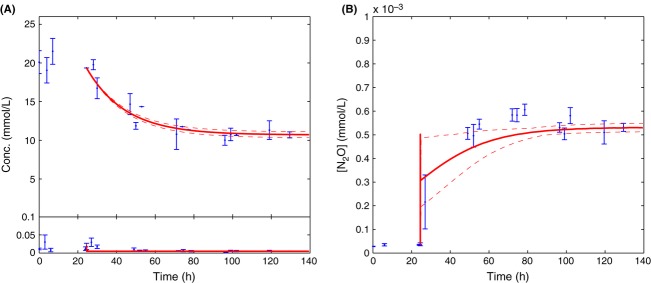
Comparison between the model and the high-copper results of Felgate et al. (2012). (A) The upper and lower panels show 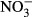 and 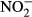, respectively. The vertical axis is continuous with different scales used in the two panels. (B) N_2_O. Experimental points have error bars. Model predictions and error bounds are shown as solid and dashed lines, respectively. The final experimental points are the pooled points.

**Figure 2:**
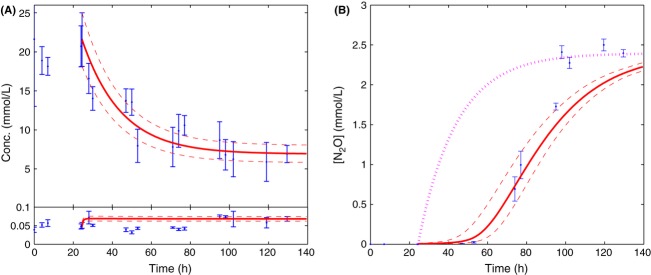
Comparison between the model and the low-copper results of Felgate et al. (2012). (A) The upper and lower panels show 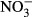 and 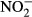, respectively. The vertical axis is continuous with different scales used in the two panels. (B) N_2_O. Experimental points have error bars. Model predictions and error bounds are shown as solid and dashed lines, respectively. The dash-dotted line in (B) corresponds to a constant Nos concentration throughout. The final experimental points are the pooled points.

The calculated enzyme levels given in Table 2 are then used to compute the time evolution of 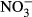, 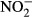, and N_2_O. The time courses for the experimental data and the model prediction are shown in Figures 1 and 2 for the high-copper and the low-copper, respectively. As can be seen from Figure 1, the model produces a good approximation to the decreasing concentration of 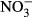 and its progression to steady state. The time courses for 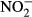 and N_2_O show that the concentrations rapidly move to their steady-state values of around 5 μmol/L and 0.5 μmol/L, respectively. The increase in the experimental 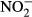 concentration at the first point after the start may be as a result of the delayed Nir expression (Baumann et al. 1996). The sharp initial change in the N_2_O concentration is due to the cumulative effect of setting the Nar, Nir, and Nor levels to their steady-state values at the start of the anoxic period. The experimental data and the kinetic parameters were varied to the limits of their error bounds to calculate the variability in the time courses, and these limits are displayed as dashed lines in Figure 1. In each case, we see that the majority of the experimental data points fall within the curves. The experimental error of the points that lie outside the bounds are incident with the curves in all but a few cases for the three time courses shown. The points which lie outside the envelops are predominantly close to the start time, which is most likely due to differences between the enzyme concentrations during the early stages of anoxia and those attained at steady state.

The time courses for 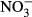, 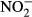, and N_2_O for the low-copper experimental data and model predictions are shown in Figure 2. The figure shows that the model produces a good approximation to the decreasing concentration of 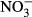 and its progression to steady state. Only one point and its error bound lie entirely outside of the error envelop for 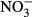. The level of 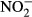 adjusts quickly to its steady-state concentration of around 0.07 mmol/L, but overestimates the 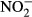 level between 40 and 80 h when the system is not at steady state. This could occur because more Nir is present than at steady state. The experimental results for N_2_O show that the emission of a significant amount of N_2_O lags behind the switch to anoxia by around 30 h. Figure 2(B) shows the N_2_O experimental results and two time courses calculated by the model. The curve that increases steeply from *t* = 24.5 h is the prediction when the Nos level is set to the computed steady-state value for the duration of the simulation. It attains the steady-state N_2_O concentration, as expected, but exhibits markedly different behavior until *t* ≈ 80 h. In contrast, the solid curve that better approximates the transient behavior is obtained by computing the initial Nos level and then decreasing it to the steady-state value using the method described in the *Experimental Procedures* section. The time course does go on to reach the steady-state N_2_O concentration, but does not reach the level within the time frame shown. However, the time-dependent Nos concentration does produce a prediction that is closer to the low-copper experimental results throughout the whole time course.

In a separate investigation, we set the Nos level to the steady-state concentration throughout the simulation and adjusted the kinetic constants for Nor to see if they could account for the lag. We found that the N_2_O emission lag could not be produced by changing K_M,4_ even by several orders of magnitude. However, the time evolution of N_2_O could be fairly well approximated when the Nor concentration is set to around 8% of its steady-state value for the duration of the lag period (*t* < 60 h). However, this results in levels of cytotoxic NO rising to around 0.1 mmol/L. Even though the toxic level of NO is unknown (Bakken et al. 2012), this level is likely to decrease cell viability (Mills et al. 2008). Therefore, it seems unlikely that the level and/or efficacy of Nor is solely responsible for the observed lag.

### Predictive analyses

In the absence of experimental data, we can use the model to predict the metabolite time courses for a range of conditions. We determine the Nos concentration from the copper level using the formulae given in the *Experimental Procedures* section. For Nar, Nir, and Nor we use the midpoints of the enzyme ranges given in Table 1 and the range limits when calculating an error estimate. By varying the copper concentration, and hence the level of active Nos, we can quantitatively predict the effect that copper has on the emitted level of N_2_O.

**Figure 3:**
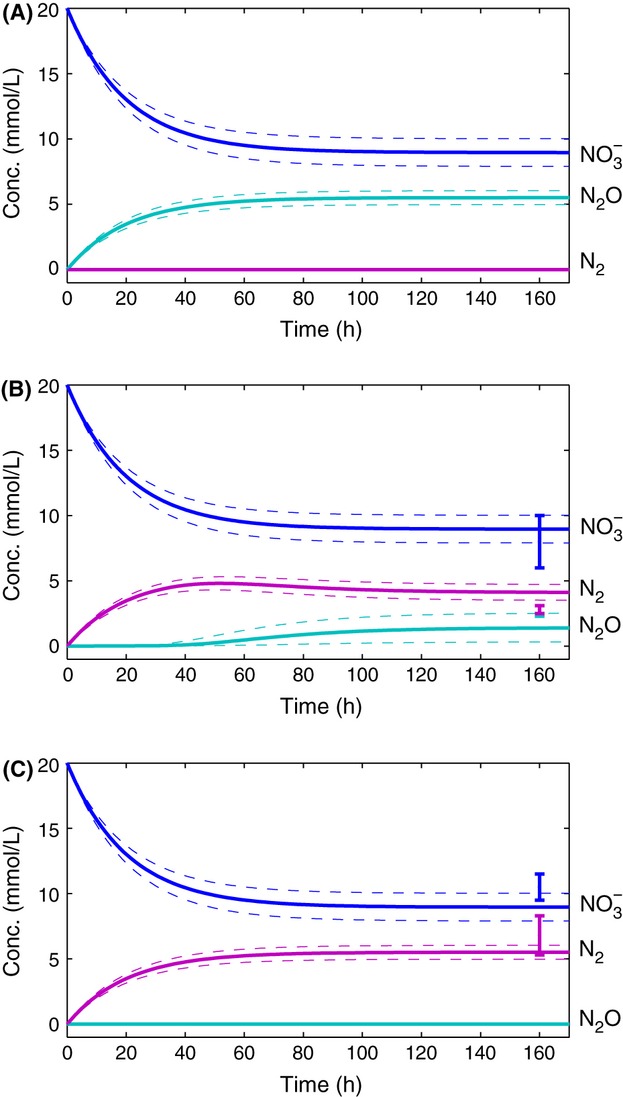
Model predictions for *n*_1,In_= 20 mmol/L and D = 0.05 h^−1^. (A) [Cu] = 0 μmol/L. (B) [Cu] = 0.5 μmol/L. (C) [Cu] = 13 μmol/L. Error bars in (B) and (C) are from Table 1 of Felgate et al. (2012).

Figure 3 shows the results from simulations performed with a dilution rate of 0.05 h^−1^, an influent 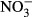 concentration of 20 mmol/L and copper concentrations of 0 μmol/L, 0.5 μmol/L, and 13 μmol/L. At *t* = 0 we set the metabolite concentrations to zero except for 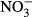 which is set to the influent concentration, which satisfies the N-mass balance. The concentration of 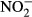 remains well below 0.1 mmol/L and the NO concentration remains at around (the prescribed) 5 μmol/L for the duration of the predictions, and so their time courses are omitted from the figures.

Figure 3A shows the time courses when [Cu] = 0 μmol/L. In this case, N_2_O is emitted straightaway and the steady-state concentration (5.51 mmol/L) is a maximum as no N_2_ is emitted. Figure 3B and C show the computed time courses together with the steady-state values quoted in Table 1 of Felgate et al. (2012), which are displayed as error bars at *t* = 160 h. In Figure 3B, where [Cu] = 0.5 μmol/L, the model over-estimates the N_2_ level but the 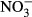 and, importantly, the N_2_O levels are incident within the error bounds. As described in the *Experimental Procedures* section, these predictions should be treated as the copper-limited case. The predictions for [Cu] = 13 μmol/L shown in Figure 3C are a good match to the experimental steady-state concentrations for 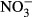 and the N_2_. The computed steady-state N_2_O level is below 1 μmol/L, which is in good agreement with the experimental value. The results shown in Figure 3B and C accurately mirror the experimental observations of Felgate et al. (2012) and demonstrates how N_2_O emissions, as predicted by the model, increase as the copper level is decreased.

## Discussion

The denitrification pathway, 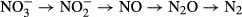, has been modeled for the growth of *P. denitrificans* under anaerobic and 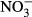-sufficient conditions in a chemostat. The kinetics-based model described here is novel on three main fronts: (1) For the first time, to our knowledge, the chemical reactions and the experimentally determined kinetic parameters are brought together for all four reductases of *P. denitrificans* and then used in a kinetic model for denitrification. (2) Enzyme expression levels and the emissions (and error bounds) of the metabolites in the denitrification pathway are quantitatively predicted. (3) The impact of copper on the Nos reductase is included, and hence its effect on N_2_O emissions. The results presented herein could ultimately help shed light on the macroscopic emission of N_2_O due to denitrifying bacteria at low-copper levels.

The rate of change in each nitrogenous compound's concentration is described by a set of coupled ODEs. Competition and inhibitory effects (e.g., Kǔcera 1992) are omitted from the model in its present form, but they could be included into the set of ODEs, if required. The model focuses on the model denitrifier, *P. denitrificans*, which expresses the four enzymes required by the pathway. Experimentally determined kinetic parameters for each enzyme are collated from the literature, displayed in Table S1 and used by the model. When experimental data are available, enzyme levels can be deduced from simple algebraic equations. Without experimental data, predictions are made using enzyme levels calibrated from published experimental results.

The nitrate-sufficient experimental results of Felgate et al. (2012) for low- and high-copper levels are used to calibrate the model. The calculated steady-state levels for 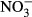 reductase (Nar), 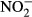 reductase (Nir), and NO reductase (Nor) are similar for the two copper levels. However, the implied N_2_O reductase (Nos) level is found to be 10–30 times less in the low-copper experiment. A difference in regulation is expected due to copper dependence of Nos, but here we have predicted the factor. A rectangular hyperbola is chosen to model the copper dependence of Nos and it is calibrated from experimental datasets via implied enzyme concentrations. In reality the Nos level should decrease at high-copper levels due to cytotoxic effects (Banci et al. 2010). The Nos level prediction formula forms a ‘piece’ of the model and it is used to provide the fourth and final enzyme concentration required when making predictions. It should be noted that a future investigation into the copper dependence of Nos for a full range of copper concentrations would improve the calibration of this curve.

The model can predict the time courses of the denitrifying intermediates when the enzyme levels, the flow conditions and the initial conditions are given. These time courses are shown to accurately predict the experimentally observed time courses, subject to the error bounds of the experimental data and the kinetic parameters. Most importantly, transient and steady-state levels of the potent greenhouse gas, N_2_O, are predicted. It is important to point out that constant enzyme levels are used for Nar, Nir, and Nor throughout the time-course calculations. By contrast, the Nos concentration is given a time dependence because of an observation made by Felgate et al. (2012). In their low-copper experiment the N_2_O level did not become significantly high for around 50 h after the switch to anoxia. The initial pool of Nos is therefore more catalytically active and able to reduce N_2_O than at a later time. If this lag is attributed solely to the function of the Nos enzyme, then it seems that the initial apo–Nos population readily acquires the requisite 12 copper atoms to form fully functional Nos. However, at a later stage, as the duration of anoxia increases, this ability becomes compromised. Interestingly Baumann et al. (1996) observed that *P. denitrificans* rapidly expresses the gene for Nos during the switch to anoxia, with the expression level peaking before declining to the steady-state level. This increased likelihood of apo-Nos forming fully functional Nos in the very early stages of anoxia is modeled here by setting the initial Nos concentration to a value calibrated from the predictions for the experimental data. The Nos level is decreased to the steady-state value exponentially using the dilution rate, as if chemostat washout was the cause of the concentration change. As a result, the calculated N_2_O time courses acceptably match the experimental data although the low-copper prediction underestimates the level on its way to the steady-state concentration, indicating that the concentration probably changes more suddenly.

Apart from a gradual change in the Nos level, there could be several other explanations for the N_2_O emission lag. In the low-copper experiment of Felgate et al. (2012) there is a sudden increase in the amount of 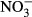 consumed at around *t* = 50 h. This would lead to a concomitant jump in the N_2_O level, which possibly exhausts the catalytic ability of the Nos pool. Alternatively, if a subpopulation of cells have a relatively high level of copper during the first stages of anoxia, perhaps due to poor mixing, those cells would be able to express fully active Nos. A decrease in this population over time would account for the lag. The lag may be a facet of the 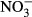-sufficient succinate-limited experiment because it is not observed in the 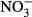-limited succinate sufficient experiment of Felgate et al. (2012). It should be pointed out, however, that the steady-state 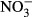 consumption in the 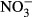-sufficient experiment is practically double that of the 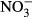-limited experiment. The Nos pool is therefore able to reduce N_2_O until a critical N_2_O concentration is reached – a level that is not exceeded in the 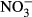-limited experiment. Finally, if experimentally determined time-dependent enzyme levels became available a suitable function could be fitted to them and used by the model.

Felgate et al. (2012) report the cell copper content to be ∼700 and ∼100 nmol g^−1^ for the high- and low-copper experiments, respectively. As the dry mass in both experiments is ∼0.2 g L^−1^, these values represent 1 and 4% of the total copper present in the reactor reservoir (∼13 and ∼0.5 μmol/L, respectively) and includes the amount of copper in all of the copper-dependent enzymes. However, these percentages do not take into account the bioavailability of reservoir copper, which for the low-copper experiment is anticipated to be substantially less than 0.5 μmol/L due to the use of ascorbate and copper-chelator BCA in the medium. Indeed, both values indicate that the majority of the copper remains extracellular. The Nos concentrations predicted by the model for the experiments are 3.32 nmol/L (high-copper) and 0.23 nmol/L (low-copper) that represent less than 1% of the total copper (assuming 12 copper atoms per Nos dimer), which is consistent with Felgate et al. (2012). Furthermore, these values estimate the amount of copper in the active Nos population only.

As the model considers a single bacterium, *P. denitrificans*, it would only form a component of a model describing a chemostat containing a mixed culture. If similar models were available for other bacteria then an ensemble model for a mixed bacterial community could also be created, although any competition and inhibition between those bacteria would have to be included. The model could, however, be easily adapted to another bacterium provided the configuration of the denitrification pathway and specific kinetic parameters were available. The experiments used for calibration were performed at pH 7.5 and so any significant variation from this value would challenge the applicability of the kinetic constants and hence the veracity of the predictions. Furthermore, acidic pH levels were found to hinder Nos formation (Bakken et al. 2012). Nonetheless, the results described here could ultimately help shed light on the macroscopic emission of N_2_O due to *P. denitrificans* at low copper levels, as might be found in those areas of agricultural land where the bioavailability of copper is limited.

## Supporting Information

**Table S1.** Kinetic parameters and molecular weights for the reductases of *P. denitrificans*. Each value was obtained at a pH of around 7 and a temperature between 25°C and 35°C inclusive. When the error bound is not stated in the referenced article an error bound of 20% is used. Unit conversions from μmol/L min^−1^mg^−1^ to sec^−1^ are performed by multiplying the activity by the enzyme weight and dividing by 60.

**Table S2.** Steady-state concentrations for the high- and low-copper experiments of Felgate et al. (2012) computed from all points lying in the time ranges stated therein. The NO concentration is prescribed to be half the experimental detection limit of 10μmol/L, and the error allowed to be 20%.

**Table S3.** Calibration constants for the Nos prediction function (eq. 8).
