# Supplementary material for: Modeling the effect of copper availability on bacterial denitrification
Source: Microbiologyopen. 2013 Jul 30;2(5):756–65. doi: 10.1002/mbo3.111 (PMC3831637; doi:10.1002/mbo3.111)
Supplement: Supplementary file 1 [file mbo30002-0756-SD1.docx]

**Table S1**: Kinetic parameters and molecular weights for the reductases of *P. denitrificans*. Each value was obtained at a pH of around 7 and a temperature between 25°C and 35°C inclusive. When the error bound is not stated in the referenced article an error bound of 20% is used. Unit conversions from μmol min^−1^mg^−1^ to s^-1^ are performed by multiplying the activity by the enzyme weight and dividing by 60.

| **Enzyme** | **Parameter** | **Value** | **Reference** |
| --- | --- | --- | --- |
| **Nar** |  |  |  |
|  | K_M_ | 13 μM | Craske and Ferguson, 1986 |
|  | Activity | 50 μmol min^−1^mg^−1^ | *Ibid.* |
|  | Weight | 209 kDa | *Ibid.* |
|  | K_cat_ | 174 s^−1^ |  |
| **Nir** |  |  |  |
|  | K_M_ | 12±2.4 μM | Richter *et al*., 2002, Table II |
|  | K_cat_ | 74±12.5 s^−1^ | *Ibid.* |
| **Nor** |  |  |  |
|  | K_M_ | 35 μM | Thorndycroft *et al*., 2007 |
|  | K_cat_ | 4877 min^−1^ | *Ibid.* |
| **Nos** |  |  |  |
|  | K_M_ | 6.7 μM | Snyder and Hollocher, 1987 |
|  | Activity | 122 μmol min^−1^mg^−1^ | *Ibid.* |
|  | Weight | 130 kDa | Haltia *et al*., 2003 |
|  | K_cat_ | 264 s^−1^ |  |

Craske, A., and S. J. Ferguson. 1986. The respiratory nitrate reductase from *Paracoccus denitrificans*. Eur. J. Biochem. 158:429–436.

Haltia, T., K. Brown, M. Tegoni, C. Cambillau, M. Saraste, K. Mattila, et al. 2003. Crystal structure of nitrous oxide reductase from *Paracoccus denitrificans* at 1.6 Å resolution. Biochem. J. 369:77–88.

Richter, C. D., J. W. A. Allen, C. W. Higham, A. Koppenhöfer, R. S. Zajicek, N. J. Watmough, et al. 2002. Cytochrome cd1, reductive activation and kinetic analysis of a multifunctional respiratory enzyme. J. Biol. Chem. 277:3093–3100.

Snyder, S. W., and T. C. Hollocher. 1987. Purification and some characteristics of nitrous oxide reductase from *Paracoccus denitrificans*. J. Biol. Chem. 262:6515–6525.

Thorndycroft, F. H., G. Butland, D. J. Richardson, and N. J. Watmough. 2007. A new assay for nitric oxide reductase reveals two conserved glutamate residues form the entrance to a proton-conducting channel in the bacterial enzyme. Biochem. J. 401:111–119.
