# Supplementary material for: Modeling the effect of copper availability on bacterial denitrification
Source: Microbiologyopen. 2013 Jul 30;2(5):756–65. doi: 10.1002/mbo3.111 (PMC3831637; doi:10.1002/mbo3.111)
Supplement: Supplementary file 2 [file mbo30002-0756-SD2.docx]

**Table S2:** Steady-state concentrations for the high- and low-copper experiments of Felgate *et al*. (2012) computed from all points lying in the time ranges stated therein. The NO concentration is prescribed to be half the experimental detection limit of 10 μM, and the error allowed to be 20%.

| **Experiment** | **[NO_3_^-^]** | **[NO_2_^-^]** | **[NO]** | **[N_2_O]** |
| --- | --- | --- | --- | --- |
| High-copper | 10.71±0.39 mM | 4.95±0.87 μM | 5±1 μM | 0.53±0.02 μM |
| Low-copper | 6.91±1.10 mM | 68.50±6.29 μM | 5±1 μM | 2.39±0.05 mM |
