# Supplementary material for: Modeling the effect of copper availability on bacterial denitrification
Source: Microbiologyopen. 2013 Jul 30;2(5):756–65. doi: 10.1002/mbo3.111 (PMC3831637; doi:10.1002/mbo3.111)
Supplement: Supplementary file 3 [file mbo30002-0756-SD3.docx]

**Table S3:** Calibration constants for the Nos prediction function (equation (8)).

| **Equation** | **α (nM)** | **β (μM)** |
| --- | --- | --- |
| Steady-state conc. (e_4,ss_) | 7.94 | 17.80 |
| Steady-state conc. - upper bound | 7.79 | 15.14 |
| Steady-state conc. - lower bound | 8.55 | 22.61 |
| Initial conc. (e_4,Init_) | 7.94 | 4.64 |
